# The Role of Fermentable Fibre on Endurance Exercise Capacity: A Randomised Crossover Trial of Inulin Supplementation

**DOI:** 10.1111/nbu.70010

**Published:** 2025-05-21

**Authors:** L. Torquati, H. Power, T. Pons, J. Bowtell

**Affiliations:** ^1^ Public Health and Sport Sciences, Medical School University of Exeter Exeter UK; ^2^ Natural Sciences, Faculty of Environment, Science and Economy University of Exeter Exeter UK

**Keywords:** acetate, exercise performance, inulin, microbio*, muscle perfusion, time trial

## Abstract

Manipulation of the mouse gut microbiome has been shown to increase gut‐derived short‐chain fatty acids and improve exercise capacity. Associations between exercise performance and gut microbiome composition and metabolites have also been identified in human studies. Yet there is little direct evidence that prebiotics are able to increase acetate production and improve exercise capacity in human participants. We conducted a randomised controlled cross‐over trial with 21 healthy and active males (35.0 ± 6.9 years; 24.4 ± 2.7 kg/m^2^) to investigate the effect of 15 g of inulin (prebiotic) on exercise performance (15 km cycle time trial), compared to placebo. Time to completion of a 15 km time trial was the primary outcome, while plasma acetate concentration and markers of inulin fermentation (breath H_2_ concentration) and muscle oxygenation were measured to explore potential mechanisms of action. Time to complete the 15 km time trial was not affected by inulin mean difference between inulin and placebo trials: (−10.37 s, 95% CI [−150.8, 130.1 s], *p* = 0.884). The marker of inulin fermentation (H_2_ concentration increase from baseline) was significantly higher in inulin compared to placebo condition (+42.61 ppm, 95% CI [30.04, 55.19], *p* = 0.001 and +31.13 ppm, 95% CI [3.73, 58.51], *p* = 0.029, respectively), but plasma acetate concentration did not differ between conditions. Likewise, markers of muscle oxygenation were not different between inulin and placebo. Our current results do not support the acute use of prebiotics to improve exercise performance in adults. Possible explanations for the absence of ergogenic effects may be that the timing between prebiotic ingestion and exercise was too short to allow for complete fermentation into acetate, participants were in a fasted rather than a fed state, or that the single dose of supplement was insufficient. These factors, together with advanced methods (stable isotope studies) should be investigated in a follow‐up study to elucidate the fate and role of colonic‐derived acetate during exercise.

## 1 Introduction

Recent studies suggest a role for the gut microbiome metabolites in exercise endurance (Zhao et al. 2018; Scheiman et al. 2019). Mechanisms proposed include that microbial‐derived short‐chain fatty acids (SCFA) provide an alternative fuel for muscle during exercise (Zhao et al. 2018; Scheiman et al. 2019; Ismaeel et al. 2023) and/or increase muscle perfusion (Okamoto et al. 2019). Both these putative mechanisms identify acetate as a metabolite potentially supporting exercise endurance. Reductions in acetate production achieved via antibiotic depletion of the gut microbiome resulted in reduced exercise capacity in mice (Okamoto et al. 2019). This was reversed by restoring the gut microbiome with a faecal transplant and feeding fermentable fibre, which increased faecal acetate levels and exercise endurance compared to baseline (Okamoto et al. 2019).

Cross‐sectional studies show that athletes' gut microbiome and SCFA production are different to that of sedentary people (Barton et al. 2018), and that aerobic capacity (VO_2_ peak) was positively associated with higher microbial diversity and higher short‐chain fatty acid production (Estaki et al. 2016). Even acute exercise induces significant shifts in bacterial species and metabolic pathways, as observed after participants completed a half‐marathon (Zhao et al. 2018). Recent reviews on physical activity and exercise associations with gut microbiome (Mitchell et al. 2019; Ortiz‐Alvarez et al. 2020; Aya et al. 2021; Dziewiecka et al. 2022) highlight the dearth of longitudinal exercise training studies in athletes, as most intervention studies included physical activity interventions in sedentary and/or overweight adults (Allen et al. 2018; Munukka et al. 2018; Kern et al. 2020; Rettedal et al. 2020). These studies showed that 6 weeks of exercise significantly shifted bacterial species and increased colonic SCFA production in sedentary or overweight adults. Studies in athletes showed an increase in SCFA‐producing species following both acute intensive exercise (Zhao et al. 2018; Tabone et al. 2021) and after 3–4 weeks of training (Keohane et al. 2019; Craven et al. 2022). Interestingly, one study (Hampton‐Marcell et al. 2020) found that reducing peak swim training volume during the competitive season training taper period coincided with a reduction in alpha diversity and a 43% shift in beta diversity (Bray‐Curtis distance). Evidence from human and mice studies (Choi et al. 2013; Evans et al. 2014) consistently shows that exercise increases gut bacterial diversity and abundance of SCFA producers, but so far no study has proven a specific mechanism to explain these outcomes.

The SCFA suggested to mediate the observed relationship between gut microbiome and exercise performance are acetate and propionate (demonstrated in humans), and butyrate in mice. These metabolites are normally produced through the fermentation of carbohydrates that escape digestion (dietary fibre, resistant oligosaccharides), with acetate being the predominant SCFA produced with the highest systemic availability (van der Beek et al. 2018). Manipulation of SCFAs through dietary supplementation is well characterised (Gibson et al. 2017; Le Bastard et al. 2020). Inulin is a resistant oligosaccharide shown to mainly increase acetate production in the colon (measured in faeces) after both acute and chronic ingestion (Boets et al. 2015; Rahat‐Rozenbloom et al. 2017; van der Beek et al. 2018; Le Bastard et al. 2020). Following production, acetate is transported through the portal vein to the liver, to subsequently reach systemic circulation (Bloemen et al. 2009).

A series of acute studies using 15–24 g of inulin with/without infusion of ^13^C‐labelled SCFAs quantified the in vivo SCFA production and absorption in the colon, and resulting systemic concentrations (Boets et al. 2015, 2017; van der Beek et al. 2018). A 15 g inulin acute dose, alongside primed continuous intravenous infusion of ^13^C‐labelled SCFA for up to 7 h post‐ingestion, reported a colonic‐derived acetate rate of appearance in plasma of 13.3 ± 4.8 mol kg^−1^ min^−1^ (Boets et al. 2015). In a similar study ^13^C labelled SCFA delivered with gastro‐resistant capsules directly into the colon (200 mg of ^13^C‐labelled sodium acetate, 170 mg of ^13^C‐labelled sodium propionate, or 495 mg of ^13^C‐labelled sodium butyrate) (Rahat‐Rozenbloom et al. 2017) resulted in acetate increase from 3 h and peak at ~6 h post‐ingestion. The resulting systemic availability of SCFA was reported as 36% acetate, 9% butyrate, and 2% propionate; since most of these SCFA were excreted in lungs as CO_2_ and only < 6% were incorporated into glucose (van der Beek et al. 2018). Finally, in overweight and obese men, an acute dose of 23.5 g inulin + 0.5 g U‐^13^C‐inulin, delivered in high‐fat milkshake (~550 kcal, 46% fat/42% carbohydrates/12% protein) resulted in increased plasma ^13^C‐acetate and exhaled CO_2_ enrichments from 2 h post‐consumption. Plasma acetate during the 7 h test period was higher compared to placebo (iAUC 0–7 h: 1310 ± 522 vs. −10 169 ± 834 μmol/L·7 h, *p* = 0.007, respectively). Collectively, these studies demonstrated that acutely ingested inulin is fermented in the colon and the resulting acetate (the most abundant SCFA) can cross the gut epithelial barrier and increase systemic acetate concentrations in humans within 2–4 h post‐consumption. Nevertheless, the effect of such elevations in acetate production following inulin or other fermentable carbohydrates supplementation on exercise performance has not yet been investigated.

In mice, manipulation of acetate systemic concentrations alters exercise endurance (Okamoto et al. 2019). A reduction in colonic acetate production (by depleting gut microbiome and removing fermentation substrate—i.e., dietary fibre) resulted in reduced time to exhaustion in mice. When the gut microbiome was restored and dietary fibre re‐introduced to diet, acetate production increased resulting in higher running time to exhaustion compared to control (Okamoto et al. 2019). Acetate may play a role in energy metabolism during exercise, as previous acetate colonic infusion increased energy metabolism and fat oxidation in human participants (Canfora et al. 2017). However, there is currently no direct in vivo human evidence that colonic acetate is directly metabolised by muscle during exercise independent of hepatic sources (Evans et al. 2001; Aoi et al. 2023; Ismaeel et al. 2023). It is possible that multiple mechanisms contribute to the apparent relationship between exercise performance and gut microbial composition and metabolites. For instance, in addition to potentially providing an alternative fuels source, SFA may enhance muscle perfusion.

Acetate ingestion (100 mL/day, 4%–5% acetic acid) has been shown to enhance flow‐mediated vasodilatation compared to placebo in a 3‐day cross‐over trial. In a parallel in vitro element of this same study, the addition of acetate (100–200 μL/L) to the incubation media activated endothelial cell eNOs‐synthase via AMPK phosphorylation, in a dose‐responsive manner (Sakakibara et al. 2010). In another study, acetate ingestion increased incremental flow rate in the human forearm 230 min post‐ingestion compared to placebo (Mitrou et al. 2015). This evidence, together with the observation that acetate induces dilation of isolated human colonic resistance arteries (Mortensen et al. 1990), supports the investigation of the contribution of vascular mechanisms to the relationship between gut microbiome‐derived metabolites and exercise.

In addition, given the interest in the gut‐muscle axis and the popularity of supplements targeting the gut to increase performance, evidence on supplementation effects is needed to inform both the public and nutrition practitioners. The aim of this study was to measure the effect of increasing acetate production through prebiotic supplementation (inulin) on exercise performance in recreationally active adult males. We hypothesised that acute inulin supplementation would significantly improve 15 km time trial performance due to an increase in acetate production from inulin fermentation (systemic acetate concentrations) compared to placebo, which does not contain fermentable fibre. We hypothesised increased systemic acetate will increase muscle oxygenation via increased perfusion and thus exercise performance.

## 2 Methods

### 2.1 Participants

Healthy, active males (aged 18–45 years) were invited to take part in the present randomised cross‐over trial. Participants were recruited through posters around the university campus, local notice boards, and Facebook ads. Exclusion criteria included having taken antibiotics in the past 6 months, any food allergies or intolerances, following a specific or prescribed diet from a medical professional, and currently or recently (< 4 weeks) taking probiotic and/or prebiotic supplementation. Participants gave their informed written consent to take part in this study with ethical approval from the Sport and Health Sciences Research Ethics Committee at the University of Exeter (201209‐A‐04). The participants were subsequently asked to complete a gastrointestinal symptoms questionnaire to screen for any potential conditions that could worsen following their involvement (i.e., a history of constipation, ulcerative colitis, irritable bowel syndrome, Crohns disease, or any other similar inflammatory gut condition). They also completed a questionnaire (IPAQ) to estimate habitual physical activity levels.

### 2.2 Experimental Procedure

Participants were asked to attend an initial session for completion of a maximum incremental exercise test and familiarisation with the study protocol including the time trial, followed by two testing sessions (visit 1 and 2), with at least 2 weeks washout period. This was intended to limit the training effect in between sessions and any lasting effect of the supplement. To ensure double‐blinding of investigators and participants, there was an independent researcher preparing drinks and measuring H_2_ breath during both experimental visits.

In visit 1, participants were randomly allocated to inulin condition (15 g inulin, chicory derived fructo‐oligosaccharide, Orafti Beneo ST GmbH, Mannheim, Germany) or placebo (5 g maltodextrin), and switched to the opposite condition in visit 2. Both inulin and maltodextrin were dissolved in 300 mL of water and 20 mL of no‐sugar cordial by an independent researcher, resulting in two identically flavoured and coloured drinks that were masked to both participants and the researcher. Nutritional composition for both experimental drinks is shown in Table 1.

**TABLE 1:** Nutritional composition of experimental and placebo drinks, per serve as consumed.

Component	Experimental	Placebo
Ingredients	15 g chicory root inulin^a^	5 g maltodextrin
	300 mL water	300 mL water
	20 mL cordial (sugar‐free)	20 mL cordial (sugar‐free)
Energy	22.5 kcal	20 kcal
Carbohydrates	1.2 g	5 g
Dietary fibre	13.5 g	0 g
Protein	0 g	0 g
Fats	0 g	0 g

^a^ Nutritional information from manufacturer (Orafti Inulin, Beneo) (Beneo GmbH 2024).

**FIGURE 1:**
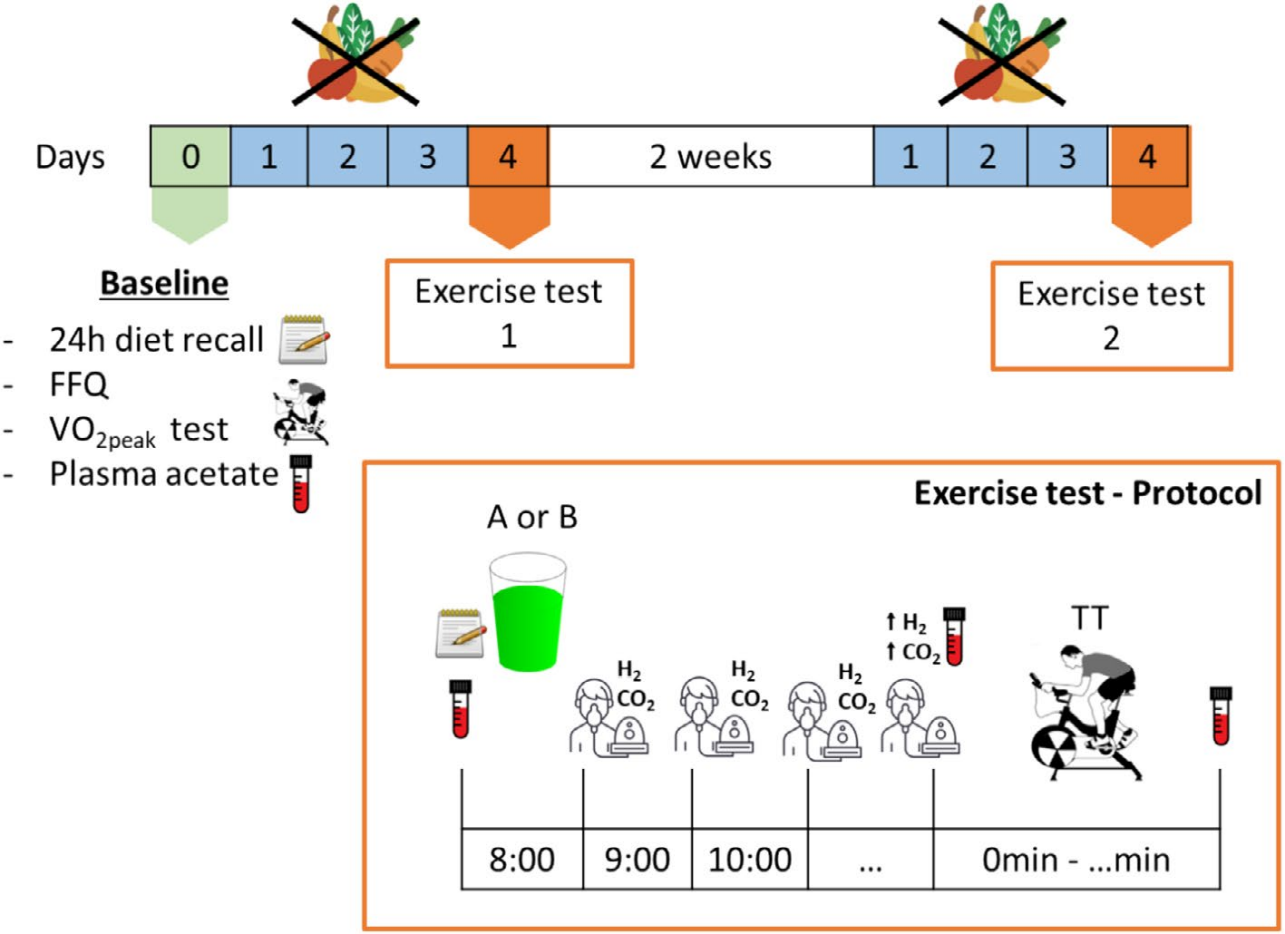
Study design schematic, showing baseline measurements, wash out period, and measurements taken at each experimental visit and respective timepoints (orange square). Arrows next to H_2_ and CO_2_ indicate significant increases in these gases' concentrations from baseline, and thus the start time for the time trial test. FFQ, food frequency questionnaire.

During the initial familiarisation session (Baseline), a sample of venous blood was collected from the antecubital vein using a vacutainer. Following this, participants completed a maximum incremental exercise test for assessment of V̇O_2 peak_ and a 15 km time trial on a Velotron bike (SRAM LLC., Chicago, IL). Participants were instructed to avoid strenuous exercise, caffeine, and alcohol in the prior 24 h; any foods or drink in the prior 12 h to the V̇O_2 peak_ test. A schematic of the study design is shown in Figure 1.

V̇O_2 peak_ was measured in an exercise test in which V̇O_2_ and VCO_2_ were assessed by breath‐by‐breath indirect calorimetry (Cortex Metalyzer 3B, Biophysik GmbH. Leipzig, Germany). The Cortex indirect calorimeter was calibrated for gas and volume prior to each test, using a reference gas (16.35% O_2_ and 4.28% CO_2_) and a 3‐L volume calibration syringe (Hans Rudolf Inc. Kansas City, MO, USA). The cycling exercise test (Monark ergometer, Cosmed srl., Rome, Italy) followed a ramp protocol (Myers et al. 1991; Coombes and Skinner 2020), which involved a 2 min unloaded warm‐up subsequently increasing exercise workload at a rate of 25 watts/min. Respiratory exchange ratio (RER), oxygen and carbon dioxide consumption were recorded throughout the test. The test was concluded when participants reached maximum effort (Borg scale) or were unable to maintain the given cadence (> 80 rpm) or reach a Respiratory Exchange Ratio (RER) > 1.2, indicating anaerobic metabolism. Exercise aerobic capacity (time‐on‐test) was calculated as the time at which the test stopped. Data was analysed with MetaSoft Studio (Biophysik GmbH. Leipzig, Germany) and V̇O_2 peak_ was defined as the mean of the highest two 30‐s epoch values (Coombes and Skinner 2020). The intensity at which the participant reached 65% of their V̇O_2 peak_ was recorded and used in experimental visits 1 and 2 as the warmup intensity prior to the 15‐km time trial.

### 2.3 15 km Time Trial

The time trial followed a validated protocol (Morgan et al. 2019), which involved participants cycling on a Velotron stationary bike (Quarq, Sram LCC, Illinois, USA) to a 15 km target. Participants were blinded to the time and distance elapsed, and were given feedback every 5 km achieved, and a final feedback with 2 km remaining to complete. Time (seconds), distance (km) and workload (watts) were recorded every 30 s using manufacturer's software and downloaded in Excel for further analysis. Time to complete 15 km was the primary measure of exercise performance, followed by average and maximum power achieved, and time to complete each 5 km split (5–10–15 km). We measured the reliability of performance outcomes by calculating the interclass correlation coefficient between baseline and placebo visit on time to completion (seconds), and mean and max power (Watts). The reliability of the time trial in measuring changes in total time was moderate (ICC 0.696, 95% CI [0.262, 0.877], *p* = 0.005), but was excellent for mean and max power (ICC 0.983, 95% CI [0.957, −0.994], *p* < 0.001 and ICC 0.926, 95% CI [0.810, 0.972], *p* < 0.001, respectively).

### 2.4 Dietary Measurements

Participants were asked to complete a food frequency questionnaire (EPIC‐Norfolk) (Bingham et al. 1997) and 24 h recall (Jonnalagadda et al. 2000) on the familiarisation visit to assess habitual dietary intake. During the 3 days leading up to experimental visits 1 and 2, participants were asked to undertake a low fibre diet (≤ 10 g/day fibre), following instructions in line with clinical practice guideline for hydrogen breath tests to standardise baseline hydrogen before supplementation (Rezaie et al. 2017; NHS West Hertforshire Hospitals 2018). This test is used as an indicator of carbohydrate fermentation. To maintain the same carbohydrate load in both experimental visits, participants were provided the same meal to be consumed the night before the visit (Tesco Frozen Mac ‘n’ cheese, 400 g. Tesco Supermarkets, UK), and asked to avoid any food or drink (other than water) 12 h before the testing session. In visits 1 and 2, participants completed a 24 h diet recall to monitor diet intake and adherence to testing guidelines on the day prior to testing. Upon arrival a blood draw was performed, and participants were then given a drink containing 15 g of inulin or placebo (2 g maltodextrin) in 350 mL of water and 15 mL of no‐sugar cordial. The allocation of drink for each trial was allocated in a pseudorandomised and counterbalanced fashion, with participants and researcher blind to condition.

### 2.5 Hydrogen Breath Analysis

Breath H_2_ was measured following manufacturers instruction and clinical guidelines (Rezaie et al. 2017; Yao and Tuck 2017). Briefly, participants were instructed to inhale using their nose, hold the breath for 10 s, and then breathe out into a mouthpiece connected to a plastic pouch (AlveoSampler) and a plastic syringe provided by the manufacturer. Once the pouch inflated, a sample of air (30 mL) was taken using the syringe to ensure alveolar air is collected. Samples were taken at baseline before drink consumption and monitored hourly post consumption for the first 3 h and then every 30 min, using BreathTracker H_2_^+^ (QuinTron Instrument Company Inc. Milwaukee, US). The time trial took place when participants' H_2_ and CO_2_ values were significantly increased. Based on (Boets et al. 2015) such increase was defined when levels were 2.5 times the standard deviation plus the running average, which is suggested to mark inulin arrival in the colon and the beginning of its fermentation into acetate. In previous studies, the appearance of acetate following a drink containing 24 g of inulin was observed between 2 and 4 h (Fernandes et al. 2011; Rahat‐Rozenbloom et al. 2017). As the placebo condition did not contain any fermentable fibre (and thus no H_2_ and CO_2_ changes from baseline) the independent researcher mocked a ‘significant increase’ in these markers at 3:30–4:00 h. A second blood draw was performed before participants began the 15 km time trial, and a third one upon completion, as described below.

### 2.6 Blood Acetate Analysis

Blood samples were withdrawn from the antecubital vein using a 5 mL lithium heparin‐coated BD Vacutainer (Benton Dickson & Co., New Jersey, USA), at baseline (first visit, familiarisation), and then at each experimental visit (before drink consumption, just before starting the time trail, after completing the time trial). After each blood collection, samples were centrifuged (10 000 rpm at 4°C for 15 min) and the obtained acellular plasma was separated and stored in 1.5 mL Eppendorf tubes at −70°C until analysis. Acetate levels were assessed with a colorimetric assay, following manufacturer's instructions (MAK086, Sigma‐Aldrich; MerkKGaA, Germany).

### 2.7 Muscle Oxygenation

Muscle oxygenation was measured with near infrared spectroscopy (Portamon, Artinis Medical Systems, The Netherlands). The device was placed over the vastus lateralis of the dominant leg. The skin was shaved and cleaned with an alcohol wipe, and the device was placed and wrapped with medical tape to secure its position. The device was then covered with a neoprene sleeve to further secure its position and avoid the influence of external light on infrared transmission, following the manufacturer's advice. Muscle oxygenation data were collected during the warm‐up and whole duration of the 15‐km time trial; outcomes included oxygenated haemoglobin, deoxygenated haemoglobin, difference in oxygenated/deoxygenated haemoglobin (%) and Tissue Saturation Index (%).

### 2.8 Participants' Feedback

After the completion of each time trial session (familiarisation, visit 1, and visit 2), participants were asked to report how they felt (‘How was this session? How was this session compared to the previous one? OR compared to the first two?’). Participants were also asked to report on the feasibility of taking part on this study, that is, how they felt following a 3‐day low fibre diet, reporting food intake, and taking part in the exercise sessions.

### 2.9 Statistical Analysis

The primary outcome measure was time to complete the 15 km time trial. In the absence of previous performance studies assessing effects of prebiotics, we based effect size on a study assessing the effect of cherry supplementation on 15 km time trial performance (*n* = 8, Morgan et al. 2019). Here, participants' mean ± SD time to complete 15 km significantly differed between placebo and treatment (1580 ± 102 and 1506 ± 86 s, respectively). Based on these differences we calculated the effect size (*d* = 0.78, converted to Coehn's *f* = 0.39) and minimum sample size (*n* = 15) to detect a difference in the main outcome. Secondary outcomes included power output (W) during the trial, blood acetate levels, breath hydrogen levels, and muscle oxygenation outcomes. Differences between groups in primary and secondary outcomes were analysed as dependent variables via one‐way ANOVA, with condition (inulin, placebo) as the independent variable. Post hoc analyses were conducted with Bonferroni adjustment and adjusted mean differences presented in results. Sensitivity analyses were conducted using habitual fibre intake (g/day) as a covariate in the above ANOVA analysis to test whether response to treatment (inulin) might depend on participants' fibre intake. Pearsons's correlations (*r* ^2^), or Spearman Rho's (*r*_*s*_) when the variables were not normally distributed, were used to explore other sources of variability.

## 3 Results

A total of *n* = 33 male participants were recruited, with *n* = 21 completing all three experimental visits. Reasons for not completing all visits included lack of time, poor health (COVID‐19 infection), conflicting schedules with competitions/training, or injury outside the experimental visits. There were no significant differences in characteristics between completers and non‐completers. The average age of participants was 32.0 ± 8.4 years, with all being classified as active based on their self‐reported physical activity levels. Participants' characteristics at baseline are shown in Table 2.

**TABLE 2:** Baseline participants' characteristics (*n* = 21, mean ± standard deviation).

Characteristic	
Mean age (years)	32.0 ± 8.4
Mean BMI (kg/m^2^)	24.4 ± 2.7
Self‐rated general health (SF‐36)	
Excellent	31.6%
Good	68.4%
Smoking Status	
Never Smoked	68.4%
Used to Smoke	26.3%
Currently Smoke	5.3%
Plasma acetate (ng/μL)	129.55 ± 43.01
Self‐reported physical activity (IPAQ)	
Mean cycling frequency (days/week)	3.3 ± 1.7
Mean session length (min)	61.6 ± 48.6
Mean session distance (km)	37.9 ± 21.5
Mean vigorous PA (min/week)	275.6 ± 250.8
Mean moderate PA (min/week)	257.5 ± 427.0
Mean V̇O2 max (mL/kg/min)	47.0 ± 9.8
Average daily nutrient intake	
Energy (kcal)	2537.6 ± 927.7
Carbohydrates (g/day)	280.1 ± 101.8
Fibre (g/day)	27.4 ± 3.2
Protein (g/day)	107.2 ± 41.1
Fat (g/day)	104.3 ± 53.1
Gastrointestinal symptoms in the previous 7 days	
Nausea/diarrhoea	0%
Gut rumbling, cramping/bloating	0%
Constipation (mild)	5.3%
Flatulence (mild)	5.3%

### 3.1 Exercise Performance—Time Trial

**FIGURE 2:**
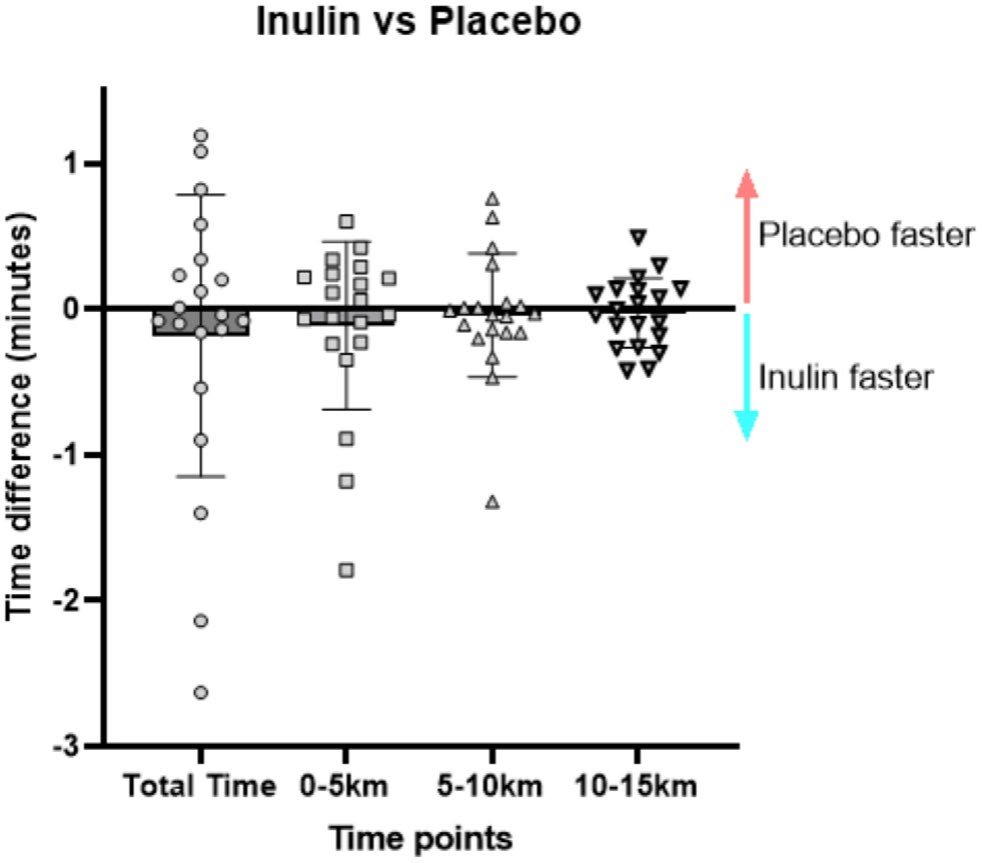
Mean time difference (in seconds) between inulin and placebo condition for total trial time and 5 km splits. Data presented as mean with bars representing standard deviation.

There were no significant differences between groups in the time taken to complete 15 km, as shown in Figure 2 (*p* > 0.05 for all outcomes). There was a large variability in the total time difference between inulin vs. placebo (−10.37 s, 95% CI [−150.8, 130.1 s], *p* = 0.884). The difference in 5 km splits was similar to total time for the first and second 5 km splits, with no significant difference between groups. When considering a meaningful difference in time trial completion of > 2% between conditions, the improvements in 15 km time trial completion were similar between conditions (6 out of 21 in inulin, 4 out of 21 in placebo, 11 out of 21 no change).

**FIGURE 3:**
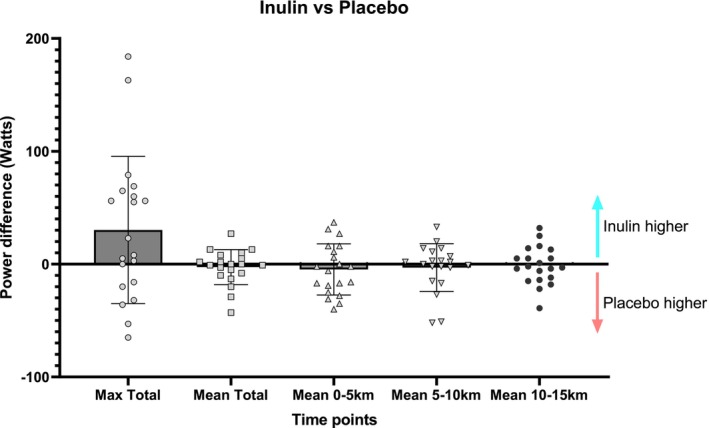
Mean power difference (in Watts) between inulin and placebo conditions for total trial time and 5 km splits. Data presented as mean with bars representing standard deviation.

There were no differences in mean and max power between conditions (Figure 3).

### 3.2 Hydrogen Breath Analysis

**FIGURE 4:**
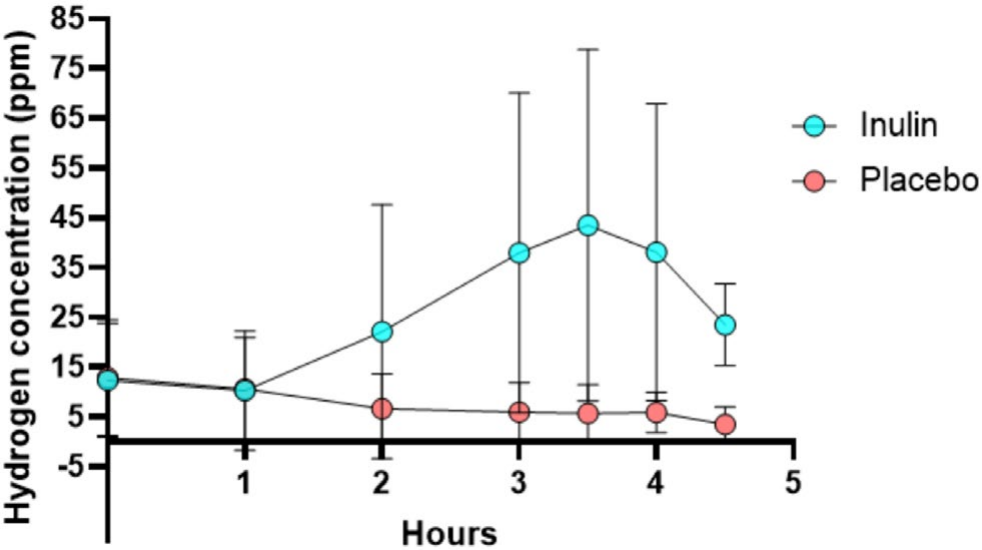
Time course of hydrogen mean concentration changes in expired air following two different experimental drinks. Data presented as mean with bars representing standard deviation.

Analysis of breath samples showed that the pattern of changes in hydrogen concentrations was significantly different between groups. There was a large variability in hydrogen concentration between participants, with the majority reaching peak hydrogen levels between 3 and 4 h timepoints. The mean difference in hydrogen concentration at these timepoints was significantly different between groups (+42.61 ppm, 95% CI [30.04, 55.19], *p* = 0.001 and +31.13 ppm, 95% CI [3.73, 58.51], *p* = 0.029, respectively) (Figure 4).

### 3.3 Acetate Changes

**FIGURE 5:**
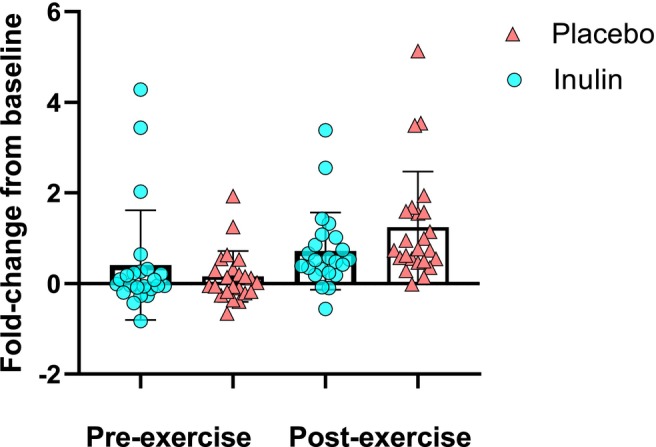
Change in acetate levels between baseline and pre‐exercise (peak H_2_ levels) and post‐exercise. Data presented as mean with bars representing standard deviation.

Plasma acetate values were measured at each testing session before the experimental drink was consumed, before the 15‐km time trial (peak H_2_ and CO_2_ levels) and after completion of the trial. There was no significant difference between changes in acetate levels between conditions (*p* > 0.05, Figure 5). The mean difference in the fold change in acetate between inulin and placebo at peak H_2_ and CO_2_ production was also not different (0.25‐fold change; 95% CI [−0.31, 0.81], *p* = 0.371), as acetate increased in both conditions. This time point is shown in Figure 5 as ‘Pre‐exercise’.

### 3.4 Muscle Oxygenation

**FIGURE A1:**
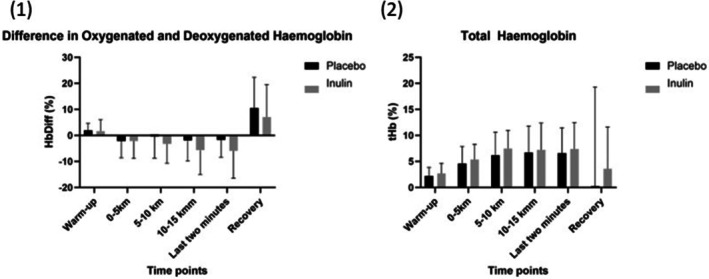
Change in the mean difference between oxygenated and deoxygenated haemoglobin percentage (1) and total haemoglobin (2) over time, with the placebo compared to the inulin supplement.

**FIGURE A2:**
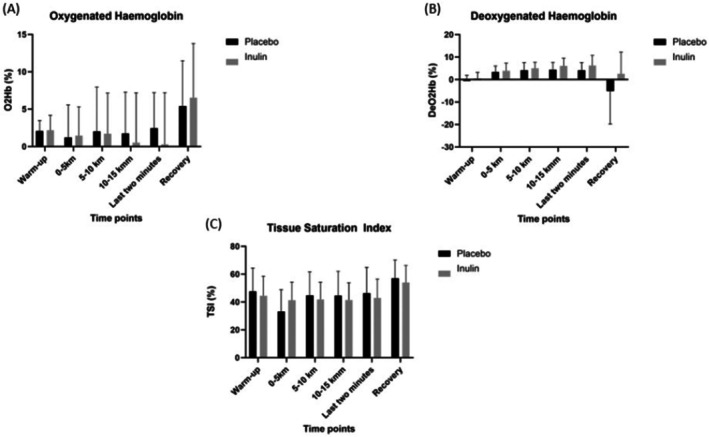
Changes in mean oxygenated (A) and deoxygenated haemoglobin percentage (B) and tissue saturation index (C) over time, with placebo and inulin supplement.

Analysis of the NIRS data showed no differences between the two conditions in muscle oxygenation outcomes. These are reported as mean differences between the two conditions during the warm‐up and at peak performance (the last 2 min) (Table 3). Differences between these markers at specific time points (i.e., 5‐km splits, average) also did not show significant differences between groups, and these are shown in Figures A1 and A2 in Appendix A.

**TABLE 3:** Mean difference of each muscle oxygenation outcome between inulin and placebo.

Parameter	Mean difference; 95% CI (inulin vs. placebo)		*p*
	Warm‐up, 65% of VO_2_	Peak performance (2 min)	
O_2_Hb (%)	0.09 [−1.25, 1.43]	−2.17 [−6.79, 2.45]	0.720
DeO_2_Hb (%)	0.35 [−1.40, 2.10]	2.07 [−1.05, 4.18]	0.418
HbDiff (%)	1.67 [−3.08, 2.54]	−5.90 [−10.7, −1.06]	0.553
TSI (%)	−3.15 [−14.8, 8.51]	−3.52 [−16.2, 9.12]	0.286
tHb (%)	0.46 [−0.964, 1.84]	0.80 [−3.08, 4.69]	0.343

### 3.5 Subgroup Analysis—Habitual Fibre Intake

Analyses on the primary and secondary outcomes were adjusted for habitual diet intake, but results were no different from the unadjusted analysis. However, we found a negative correlation between habitual diet intake (g/day) and time difference to completion between inulin and placebo (*r*_*s*_ = 0.561, *p* = 0.008). Sub‐group analysis on participants with low habitual fibre intake (< 20 g/day, based on median intake) showed a significant improvement in time to completion (−71.00 s, 95% CI [−138.75, −3.26], *p* = 0.042). Habitual dietary fibre intake did not correlate with any measures of acetate but correlated with hydrogen concentrations at 4 h post inulin consumption (*r*_*s*_ = 0.636, *p* = 0.035).

### 3.6 Participants Feedback

After the completion of each time trial session (familiarisation, visit 1 and visit 2), participants were asked to report how they felt. The majority reported feeling like they performed better in the session they had placebo, compared to the familiarisation and inulin session. But equal amounts of people in both inulin and placebo conditions reported their legs felt stronger or less tired. Overall, when asked about in which session they felt stronger or less tired, there was an equal distribution between those that reported ‘no difference between sessions’, inulin, or placebo. There were no side effects reported following a 3‐day low fibre intake, with reductions in fibre intake being significantly lower than baseline/usual intake but similar in both conditions (Inulin: 15.70 ± 2.03 g/day, Placebo: 13.84 ± 1.77 g/day, *p* = 0.326). There were no significant changes to average daily energy and nutrient intake between experimental visits (see Table A1 in Appendix A). There were no side effects associated with an acute dose of 15 g of inulin prior to exercise.

## 4 Discussion

In this cross‐over randomised controlled trial, we found no effects of prebiotic supplementation (15 g inulin) on exercise performance in healthy adult males, compared to placebo. Plasma acetate, the key gut metabolite proposed to underpin the putative ergogenic effects of prebiotics, was not significantly different between inulin and placebo. This may explain the absence of performance improvements. Yet, markers of fibre fermentation (H_2_ levels) were significantly higher in the inulin compared to the placebo condition. Like performance, markers of muscle oxygenation were not different between inulin and placebo conditions. Habitual diet intake seemed to affect the response to the supplement and positively correlated with hydrogen concentrations. Contrary to what was expected based on previous observations that higher habitual fibre intake is associated with a better response to prebiotic supplementation (Healey et al. 2018) (and hence increased acetate output and performance); in our study, habitual low‐fibre intake was associated with significant improvement in time to completion.

Our results are similar to those reporting short‐term probiotic supplementation and performance effects, but different to longer interventions. For instance, 2‐week supplementation with probiotic *Veillonela atypica* did not alter time to exhaustion at VO_2 peak_ compared to placebo (Gross et al. 2024); but longer supplementation (> 5 weeks) studies showed positive results. Five‐week supplementation with *Bifidobacterium longum* was associated with improved running distance in the 12‐min Cooper test in trained university runners (Lin et al. 2020). Supplementation with *Lactiplantibacillus plantarum* for 6 weeks resulted in improved exercise performance (VO_2_ max test duration) and reduced fatigue (Lee et al. 2022). Similar effects were observed in trained cyclists when the supplementation period was 4 months and a combination of probiotic strains was consumed (Mazur‐Kurach et al. 2022). However, none of these studies measured dietary intake or bacterial metabolites (short‐chain fatty acids, including acetate) to provide mechanistic insight into the ergogenic performance effects observed. Further, our findings on low‐fibre intake being associated with increased exercise performance with inulin supplementation contrast previous reports in the literature. Low‐fibre consumers tend to not respond to prebiotic supplementation (limited gut microbiome composition and metabolites changes) (Healey et al. 2018), which in our study seemed to be an advantage to complete 15 km time trial in a shorter time. This interesting finding warrants further investigation.

Previous studies showed performance was positively correlated with increases in SCFA producing gut microbes and with plasma SCFA, for both acute endurance exercise (marathon performance (Zhao et al. 2018), VO_2 max_ test and 1 km time trial (Tabone et al. 2021)) and endurance training interventions (Allen et al. 2018; Cronin et al. 2018; Liu et al. 2020; Hughes and Holscher 2021; Dziewiecka et al. 2022). These findings suggest a potential role of these compounds linking the microbiome and exercise performance; however, the independent contribution of SCFA to performance requires verification through further research. Although we replicated the inulin supplementation protocol shown in previous studies to enhance SCFA output (Fernandes et al. 2011; Rahat‐Rozenbloom et al. 2017) we were unable to significantly increase plasma acetate or markers of muscle perfusion. Previous evidence showed ingestion of acetic acid increased arm blood flow (Sakakibara et al. 2010; Mitrou et al. 2015), and in vitro studies show acetate can both dilate isolated colonic resistance arteries (Mortensen et al. 1990) and activate eNOS via AMPK phosphorylation in endothelial cells, in a dose–response manner (Sakakibara et al. 2010). While this evidence supported our hypothesis that acetate will enhance exercise performance via increased muscle perfusion, it is worth noting that different mechanisms have been proposed to explain the relationship between gut microbiome and exercise performance.

Evidence from mice studies showed depletion of the gut microbiome decreased gut glucose transporters Gpr41 and SGL‐1 and muscle glycogen levels (Nay et al. 2019). Thus suggesting gut microbiome modulates gut glucose absorption, muscle glucose uptake, and exercise performance (Nay et al. 2019). Yet human studies suggest either colonic acetate could be an energy substrate during exercise (Evans et al. 2001) or propionate could increase muscle energy metabolism, as both oral propionate (Chambers et al. 2018) and colonic SCFA infusions (including propionate) (Canfora et al. 2017) at concentrations that mirror those obtained with dietary fibre supplementation resulted in increased energy expenditure specifically via lipid oxidation. Given the lack of studies using methods that allow to trace the metabolic fate of ingested inulin during exercise, further research using stable isotopes is warranted to answer these outstanding mechanistic questions.

### 4.1 Strengths and Limitations

We aimed to partially replicate the manipulation of colonic acetate production previously demonstrated in mice by either removing or supplementing fermentation substrate (dietary fibre) (Okamoto et al. 2019). Our findings contrast those of Okamoto et al. (2019), who demonstrated exercise performance improved when promoting gut microbiome acetate production from fermentable fibre. The authors of that study (Okamoto et al. 2019) further confirmed these findings by increasing systemic acetate through infusions, but we only manipulated systemic acetate concentrations by increasing colonic acetate production via prebiotic supplementation. In contrast with previous literature, acetate production increased also in the placebo condition, where there was no fermentable substrate nor changes in levels of expired hydrogen. Given participants were in a fasted state and received a virtually zero calorie drink, the acetate measured in the placebo was from endogenous sources (pyruvate, liver) (Liu et al. 2018). Given the methods we used to measure acetate, we were not able to identify what percentage of the acetate produced in the inulin condition was from colonic or hepatic origin. Likewise, we were unable to measure whether acetate was taken up and utilised by muscle, impacting the plasma levels observed. A study using C^13^ labelled inulin in a fed state demonstrated that indeed ingested inulin was responsible for the increase in plasma acetate (Boets et al. 2015). Therefore, studies with labelled inulin are needed to elucidate whether the plasma acetate being measured is from endogenous or exogenous origin, whether it is taken up by muscles, and whether the origin changes compared to a fed state and/or exercise intensity.

Our results show that, before consuming the experimental drink, both groups had similar levels of hydrogen, confirming participants followed the dietary guidelines provided in line with the reported reduction in dietary fibre intake. However, our findings show that a 3‐day low fibre diet and acute 15 g of inulin are not enough to manipulate the gut microbiome to a degree that can result in measurable changes in acetate production and exercise performance outcomes. Further studies should investigate the safety of a longer period of low fibre intake (≤ 10 g/day), coupled with different doses of inulin and timing pre‐exercise to account for variability in the ability to digest inulin to produce acetate. Studies showed a range of times from 3 h (Fernandes et al. 2011; Rahat‐Rozenbloom et al. 2017) to up to 7–8 h post‐consumption to see significant production of acetate (Boets et al. 2015). As acetate production depends on specific bacterial probiotic strains that can ferment prebiotics (i.e., *Bifidobacterium*) and previous studies that used some of these probiotics showed positive exercise performance outcomes (Mazur‐Kurach et al. 2022; Lin et al. 2020; Lee et al. 2022), future studies should both measure gut microbiome composition and investigate and unpick the contribution of the substrate (prebiotic) or the live bacteria (probiotic) and whether a symbiotic supplementation would be more efficient.

The complex interaction between gut microbiome and exercise performance is a novel field, with limited evidence from mice and human studies that suggest different putative mechanisms. There are indeed different and emerging hypotheses to explain how or which gut microbiome‐derived metabolites may increase exercise performance. Current evidence in this field is mixed, with a combination of mice and human studies that so far have not provided direct in vivo evidence that clearly demonstrates the fate of colonic‐derived metabolites in specific human tissues during exercise. We aimed to contribute to the evidence base by investigating whether the previously demonstrated acetate‐induced vasodilation may improve performance via increased muscle perfusion measured in vivo during exercise. Our findings warrant further investigation using chronic supplementation and measuring gut microbiome outcomes.

## 5 Conclusion

Our results show that an acute dose of prebiotic supplement (15 g inulin) did not improve exercise performance in active adults, nor did it increase bacterial metabolites (plasma acetate) or muscle oxygenation markers. Findings warrant further investigation on the effects of chronic inulin supplementation using stratified designs to compare participants with low vs. high habitual fibre intake, with and without probiotic supplementation, in order to better understand the complex relationship between gut microbiome composition and function and exercise performance. Combination of such studies with stable isotopes infusion could help elucidate the mechanism and contribution of microbial‐derived acetate to exercise performance.

## Conflicts of Interest

The authors declare no conflicts of interest.

## Data Availability

Data available on request due to privacy/ethical restrictions.

## Appendix A

**TABLE A1:** Average daily nutrient intake across all study visits.

Average nutrient intake (±SE)	Familiarisation visit	Experimental visit—inulin	Experimental visit—placebo	*p*
Energy (Kcal/day)	2444.10 ± 210.58	2280.926 ± 184.12	2136.021 ± 135.43	0.293^a^ 0.362^b^
Carbohydrates (g/day)	279.51 ± 24.55	275.25 ± 24.86	260.10 ± 19.00	0.717^a^ 0.459^b^
Protein (g/day)	103.67 ± 9.42	97.37 ± 7.34	95.87 ± 9.37	0.567^a^ 0.810^b^
Fat (g/day)	96.97 ± 10.86	87.71 ± 10.31	77.80 ± 6.38	0.174^a^ 0.288^b^
Fibre (g/day)	27.11 ± 3.05	15.70 ± 2.03	13.84 ± 1.77	0.001^a^ 0.326^b^

^a^ Differences across all 3 visits.

^b^ Differences between intervention and placebo visits.

Figure A2 shows the change over time on muscle oxygenation outcomes, which shows there was a large variability between individuals suggesting that participants' exercise performance varied to a high extent, even within and between conditions.

Oxygenated and deoxygenated haemoglobin followed opposite trends as expected, but contrary to our hypothesis, inulin did not increase oxygenated haemoglobin compared to placebo. Quite the opposite to this, the inulin condition showed a trend for higher deoxygenated haemoglobin levels (DeO_2_Hb) with 4.01% ± 4.45% compared to 1.83% ± 4.86% in placebo. However, these differences were not statistically significant (*p* = 0.418).

Also contrary to our hypothesis, mean tissue saturation index % (marker of muscle perfusion) was not affected by inulin (47.4% ± 16.4% placebo compared to 42.6% ± 13.0% inulin, *p* = 0.286), as shown in Figure A2C.
